# Important role of precipitation in controlling a more uniform spring phenology in the Qinba Mountains, China

**DOI:** 10.3389/fpls.2023.1074405

**Published:** 2023-02-08

**Authors:** Jianhao Li, Jingyun Guan, Wangqiang Han, Ruikang Tian, Binbin Lu, Danlin Yu, Jianghua Zheng

**Affiliations:** ^1^ College of Geography and Remote sensing Sciences, Institute of Arid Ecology and Environment, Key Laboratory of Oasis Ecology, Xinjiang University, Urumqi, China; ^2^ College of Tourism, Xinjiang University of Finance & Economics, Urumqi, China; ^3^ School of Remote Sensing and Information Engineering, Wuhan University, Wuhan, China; ^4^ Department of Earth and Environmental Studies, Montclair State University, Montclair, NJ, United States

**Keywords:** Qinba Mountains, spring phenology, more uniform, elevation gradients, precipitation

## Abstract

Under global warming, the gradual pattern of spring phenology along elevation gradients (EG) has significantly changed. However, current knowledge on the phenomenon of a more uniform spring phenology is mainly focused on the effect of temperature and neglected precipitation. This study aimed to determine whether a more uniform spring phenology occurs along EG in the Qinba Mountains (QB) and explore the effect of precipitation on this pattern. We used Savitzky-Golay (S-G) filtering to extract the start of season (SOS) of the forest from the MODIS Enhanced Vegetation Index (EVI) during 2001-2018 and determined the main drivers of the SOS patterns along EG by partial correlation analyses. The SOS showed a more uniform trend along EG in the QB with a rate of 0.26 ± 0.01 days 100 m^-1^ per decade during 2001-2018, but there were differences around 2011. A delayed SOS at low elevations was possibly due to the reduced spring precipitation (SP) and spring temperature (ST) between 2001 and 2011. Additionally, an advanced SOS at high elevations may have been caused by the increased SP and reduced winter temperature (WT). These divergent trends contributed to a significant uniform trend of SOS with a rate of 0.85 ± 0.02 days 100 m^-1^ per decade. Since 2011, significantly higher SP (especially at low elevations) and rising ST advanced the SOS, and the SOS at lower altitudes was more advanced than at higher altitudes, resulting in greater SOS differences along EG (0.54 ± 0.02 days 100 m^-1^ per decade). The SP determined the direction of the uniform trend in SOS by controlling the SOS patterns at low elevations. A more uniform SOS may have important effects on local ecosystem stability. Our findings could provide a theoretical basis for establishing ecological restoration measures in areas experiencing similar trends.

## 1 Introduction

Vegetation phenology is a natural phenomenon with an annual cycle that is formed by long-term adaptation to seasonal environmental changes (Liu et al., 2014; Ganjurjav et al., 2020; Cheng et al., 2021). Such phenomena often display a clear gradual pattern with increasing elevation. For example, the dates of leaf unfolding or senescence in many places show gradual postponement or advancement (Piao et al., 2011; Wu et al., 2021). This gradual variation in phenological characteristics presents a fascinating natural landscape (Gao et al., 2019). Moreover, this gradual pattern of vegetation phenology along elevation gradients (EG) plays a key role in maintaining the stability of ecosystem structure, such as carbon and nitrogen cycling, species distribution, climate feedback, and ecosystem service functions (Cong et al., 2012; Tao et al., 2018a; Shen et al., 2020; Sun et al., 2022).

However, a under climate warming, significant changes are occurring in phenological characteristics and their interactions (Shen et al., 2014; Wolf et al., 2017; Zhang et al., 2021a). For example, the reduction in chilling units due to warming could offset the increase in forcing units, and the negative impact of a higher temperatures on the start of the season (SOS) could be counterbalanced by higher precipitation (Li et al., 2020a; Wang et al., 2021). This interaction leads to a constant or delayed a SOS in some areas (Wolkovich et al., 2012; Meng et al., 2019). There is growing concern that this progressive pattern of elevation-induced phenological shifts may be changing. For instance, Chen et al. (2018) found that the spring phenology is becoming more uniform at different elevations in Europe. Temperature is generally considered the primary control of spring phenology (Piao et al., 2006; Li et al., 2016; Tao et al., 2018b). Specifically, winter warming may reduce chilling exposure at low elevations and increase spring forcing accumulation for leaf unfolding; the low temperatures at high elevations and relative increases in effective chilling accumulation may reduce the forcing requirement (Fu et al., 2015a; Asse et al., 2018; Vandvik et al., 2018). These divergent trends of leaf unfolding between high and low elevations contribute to a more uniform spring phenology.

However, the impact of temperature on the SOS is a nonlinear process, and warming (cooling) in winter will offset the advanced (delayed) SOS caused by warming (cooling) in spring to some extent (Cong et al., 2017; Piao et al., 2019; Ettinger et al., 2020; Fu et al., 2020). The SOS patterns along EG may not be fully explained temperature alone. Furthermore, recent studies suggest that precipitation may play a key role in spring phenology (Yuan et al., 2020; Sun et al., 2021; Gong et al., 2022). Precipitation somewhat determines the light and heat use efficiency of vegetation and then affects the spring phenology. More importantly, shifts in temporal trends of precipitation may directly alter the intensity of water stress on vegetation growth and change the sensitivity of vegetation to precipitation (Li et al., 2021b; Henry et al., 2022), especially in mountainous areas with high precipitation variability. Therefore, investigation of how the temporal trends in precipitation interact with SOS patterns is urgently needed. At present, relatively few studies have examined the more uniform spring phenology phenomenon. Moreover, these studies were mainly based on the assumption that temperature plays the dominant role (Chen et al., 2018; Vitasse et al., 2018; Dai et al., 2021), neglecting the effect of the temporal trends in precipitation on spring phenology. Therefore, the impact of the temporal trends in precipitation on a more uniform SOS along EG needs to be examined in depth.

As a north-south transition zone and a large-scale east-west ecological corridor in China, the Qinba Mountains (QB) has been a hot spot for ecological change research because of its high geographic complexity, biodiversity, and climate sensitivity (Xia et al., 2019; Zhang and Liang, 2020). With the warm-dry climate, the water stress on vegetation growth may be further enhanced, which may affect the progressive pattern of spring phenology along EG in the QB. To answer this question, it is necessary to deeply explore the role of precipitation intensity (water stress) in controlling the spring phenology patterns along EG in the QB. In this study, we compared the differences in the temporal trends in SOS along EG over the period 2001-2018 based on the MOD13Q1 Enhanced Vegetation Index (EVI) dataset. Partial correlation analysis was applied to identify the main controlling factors influencing the SOS patterns along EG. We aimed to answer the following questions: (1) whether there is a more uniform SOS along EG in the QB; (2) how do temperature and precipitation control the SOS patterns along EG; (3) what is the effect of temporal trends of precipitation on the SOS patterns along EG. A more uniform SOS may cause species to migrate along EG to adapt to environmental changes. This may impact species distribution and compromise the serviceability of mountain ecosystems (Inouye et al., 2000; Lenoir et al., 2008; Wolf et al., 2017). Therefore, addressing these issues is important for understanding and predicting vegetation patterns and their ecosystem functions under climate change.

## 2 Material and methods

### 2.1 Study area

**Figure 1:**
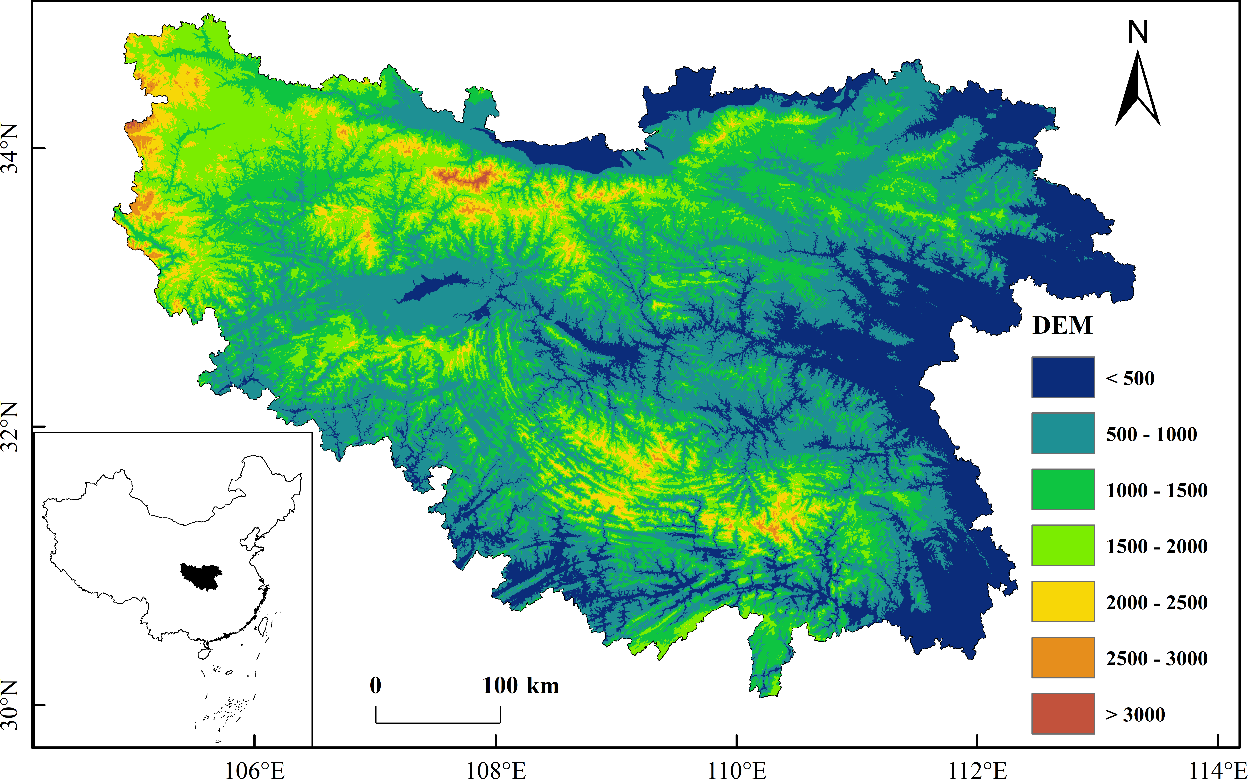
Location and elevation map of the Qinba Mountains.

The QB, which runs through Central China between 102°54′~112°40′E and 30°50′~34°59′N, is an important climatic and geographical boundary between northern and southern China (**Figure 1**). The entire region consists of three parts, namely, the Qinling Mountains, the Daba Mountains and the Jianghan Valley (Liu et al., 2016). The terrain clearly undulates, with an average annual precipitation of 700-1500 mm and an average annual temperature of 12-16 °C (Zhang and Liang, 2020). As the area is located in the transitional zone between the warm temperate zone and northern subtropical zone, vegetation types are clearly differentiated along EG, and their response to climate change is more sensitive (Deng et al., 2019; Qi et al., 2021).

### 2.2 Extraction of spring phenology

The SOS in the QB was extracted from the MOD13Q1 EVI dataset provided by the official NASA website (https://ladsweb.modaps.eosdis.nasa.gov/search/) for the period 2001-2018 with a spatial resolution of 250 m and a temporal resolution of 16 days. Due to the influence of the sensors themselves and other external factors, there were missing data or outliers, resulting in differences between the EVI time series curves and the real vegetation growth patterns (Qi et al., 2021). Therefore, we used Savitzky-Golay (S-G) filtering based on Timesat 3.3 software to eliminate noise that deviated from the normal growth trend line. By comparing the results of multiple calculations, we set the window size to 5, the envelope iterations to 2, and the adaptation strength to 8 to reconstruct the EVI time series of the QB from 2001 to 2018. Then, we defined the SOS as the date when the EVI increased to 20% of the seasonal amplitude (Wu et al., 2021). To reduce the uncertainty from the outliers, we excluded pixels with SOS earlier than the 30th day of the year or later the 180th day of the year.

### 2.3 Climate data

The Chinese meteorological forcing dataset (CMFD) is widely used for its continuous time coverage and consistent quality (http://data.tpdc.ac.cn/zh-hans/), with an accuracy between meteorological observation data and satellite remote sensing data, and a spatial resolution of 0.1° × 0.1° (Yang et al., 2010; He et al., 2020). Therefore, we selected temperature and precipitation datasets from the CMFD during 2000-2018 and analysed the temporal trends in winter temperature (WT, from the previous November to the beginning of greening in March), spring temperature (ST, from the beginning of greening in March to the end of greening in June), and spring precipitation (SP, from the beginning of greening in March to the end of greening in June) along EG in the QB at 100 m altitude intervals.

Considering the low spatial resolution of the CMFD dataset, the number of pixels in the QB is relatively small, and there may be errors based on pixel statistical analysis. To this end, we used a geographically weighted regression (GWR) model to downscale the WT, ST, and SP data to a spatial resolution of 0.01° (Lu et al., 2018; Lu et al., 2019). We selected the EVI and elevation as influencing factors and downscaled the WT, ST, and SP data by three levels with an intermediate resolution of 0.05° (**Figure S1**) (Zhang et al., 2020; Zhang and Cheng, 2020; Xu and Cheng, 2021). The values were calculated as follows:


(1) $$y_{i}=\beta_{0}\left(u_{i},v_{i}\right)+\beta_{1}\left(u_{i},v_{i}\right)x_{i1}+\beta_{2}\left(u_{i},v_{i}\right)x_{i2}+\epsilon_{i}$$

Where (*u*_*i*_,*v*_*i*_) . denotes the spatial coordinates at a spatial position *i*, *x*_*i*1_ and *x*_*i*2_ . present the values of elevation and EVI, respectively. *β*_0_, *β*_1_, and *β*_2_ . present the constant terms, the regression coefficients of elevation and the regression coefficient of EVI at raster *i*, espectively.

### 2.4 DEM and land use data

The digital elevation model (DEM) data were obtained from the 90 m resolution SRTM product provided by the Geospatial Data Cloud (https://www.gscloud.cn/). Land Use Data, derived from Globe Land Cover in 2010 (http://www.globallandcover.com/), were used to extract the distribution of the forest vegetation in the QB. The DEM and Land Use Data were resampled to 250 m to match the resolution of the EVI by using ArcMap 10.3.

### 2.5 Statistical analysis

The slope of SOS along EG for each year from 2001 to 2018 was used to compare the difference in SOS along EG among different years (**Table S1**). The smaller the slope was, the smaller the difference in SOS along EG. The quadratic curve fitting method based on Origin 2018 was used to detect abrupt changes in the slope of SOS along EG. The year corresponding to the point where the slope line of the curve intersected with the 0 slope line was considered an abrupt change point. Then, Theil-Sen trend analysis was used to analyse the temporal trends of SOS, WT, ST, and SP in the QB from 2001 to 2018 and before and after the abrupt years, and the significance of trends was tested by the Mann-Kendall (M-K) statistical test by using MATLAB R2016a (Sen, 2012; Liu et al., 2016). This value was calculated as follows:


(2) $$\beta =mean\left(\frac{x_{j}-x_{i}}{j-i}\right)\forall j>i$$

where *β* is the temporal trend of the SOS; *j* and *i* denote the time series; and *x_j_* and *x_i_* denote the SOS at times *j* and *i*, respectively. *β* > 0 indicates that the temporal trend of the SOS has a delayed trend, and *β<* 0 indicates an advanced trend.

Partial correlation analysis based on MATLAB R2016a was used to analyse the relationship between SOS and WT, ST, and SP. The degree of association between the two variables was measured by the partial correlation coefficient after excluding the effects of other control variables (Shen et al., 2020). The climate factors (WT, ST, and SP) were used as independent variables, and the SOS was the dependent variable. Statistical significance was determined at a level of *P*< 0.05 based on a two-tailed t-test. Additionally, the temporal trend and partial correlation coefficient patterns in SOS and the climate factors patterns along EG were analysed at 100 m altitude intervals. Considering the rarity of forest pixels at high elevations, regions with fewer than 100 pixels along EG were excluded, and only the regions below 3100 m were analysed in the QB.

## 3 Results

### 3.1 Spatial distribution of SOS from 2001 to 2018

**Figure 2:**
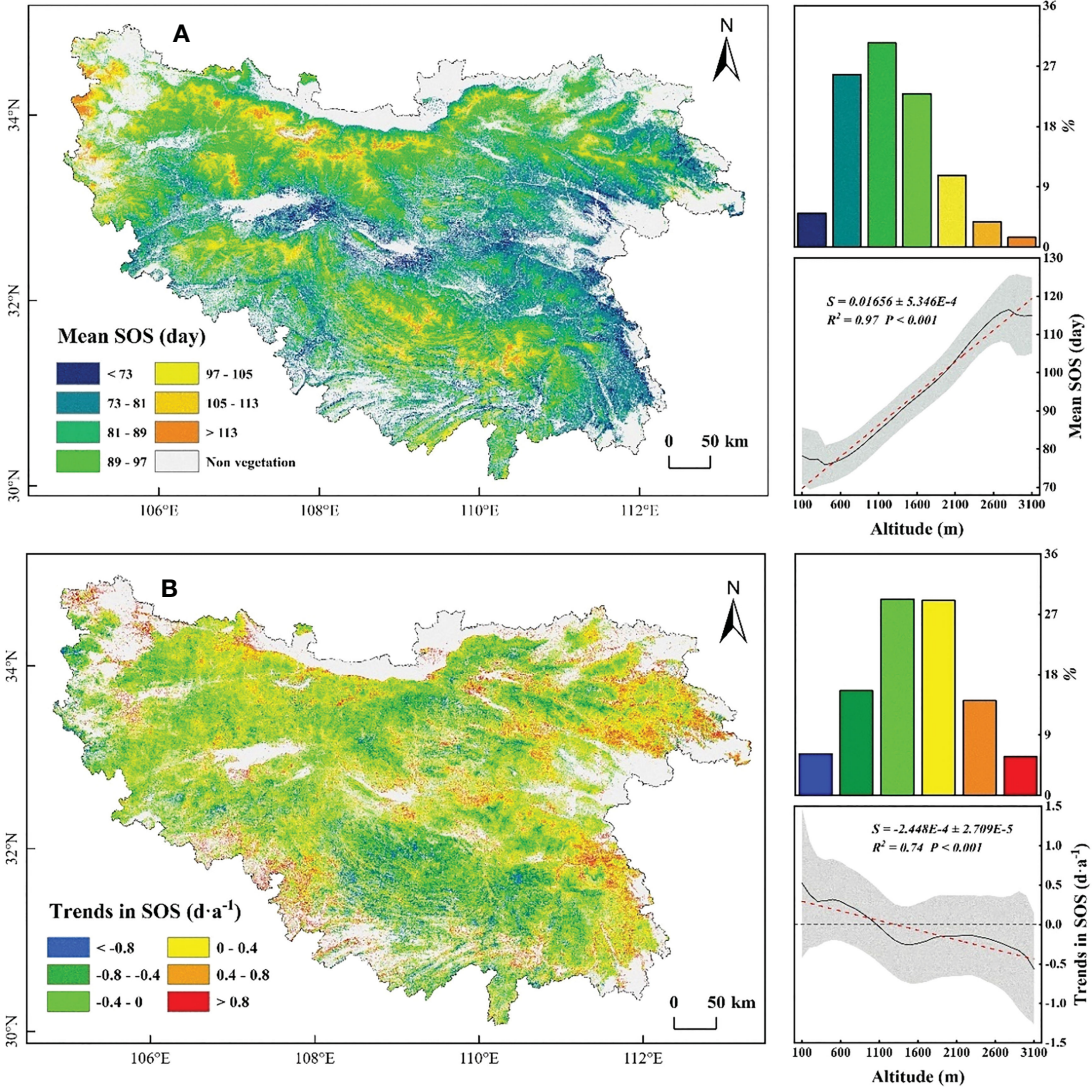
Spatial distribution of mean the start of season (SOS) **(A)** and temporal trends in SOS **(B)** during 2001-2018. The shaded area represents the standard deviation.

The mean SOS from 2001 to 2018 was early in the valleys and late on the mountains. Among them, 89.71% of the mean SOS were concentrated around 73-105 days. Along EG, the mean SOS was significantly (*P*< 0.001) delayed by 1.7 days per 100 m increase (**Figure 2A**). The temporal trends of delayed SOS (48.96% of total pixels) were mainly distributed in the valley and at marginal low elevations, and the magnitude of delay was concentrated around 0-0.8 days·a^-1^ (**Figure 2B**). Significant delayed (*P*< 0.05) were mainly distributed in the northeastern low elevation regions of the QB (**Figure S2A**). The advanced SOS (51.04%) was mainly distributed at high elevations, and the magnitude of advance was concentrated in 0-0.8 days·a^-1^ (**Figure 2B**). Significant advanced (*P*< 0.05) were mainly distributed in the western, northern, and southern alpine regions (**Figure S2A**). Along EG, the temporal trends in delayed SOS gradually decreased with increasing elevation (below 1000 m), and the advanced SOS gradually increased (above 1000 m). These divergent trends in SOS between high and low elevations showed a more uniform trend of SOS along EG in the QB.

### 3.2 Temporal trends in the SOS before and after 2011

**Figure 3:**
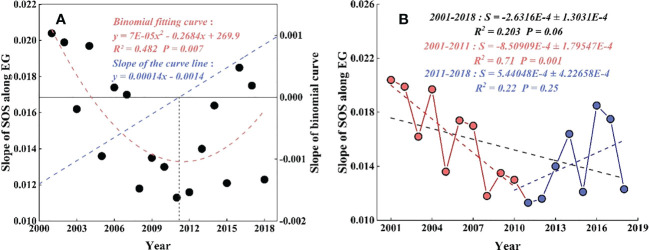
Slope change and curve slope of SOS along EG from 2001 to 2018. **(A)** The binomial curve of the slope of SOS along EG. The red line represents the binomial curve, the blue line represents the slope of the binomial curve. **(B)** The change in slope of SOS along EG from 2001 to 2018. The black linerepresents the change during 2001-2018, the red line represents 2001-2011, and the blue line represents 2011-2018.

The change in slope of SOS along EG from 2001 to 2018 decreased by 0.26 ± 0.01 days 100 m^-1^ per decade (*P* = 0.06), the differences in SOS along EG decreased continuously, and the SOS showed a more uniform trend along EG (**Figure 3B**). Furthermore, 2011 was identified as a year of abrupt change according to the change in the SOS slope along EG from 2001 to 2018 based on the quadratic curve fitting method (**Figure 3A**). The change in the SOS slope along EG significantly (*P* = 0.001) decreased by 0.85 ± 0.02 days 100 m^-1^ per decade between 2001 and 2011. Since 2011, the change was reversed by 0.54 ± 0.02 days 100 m^-1^ per decade.

**Figure 4:**
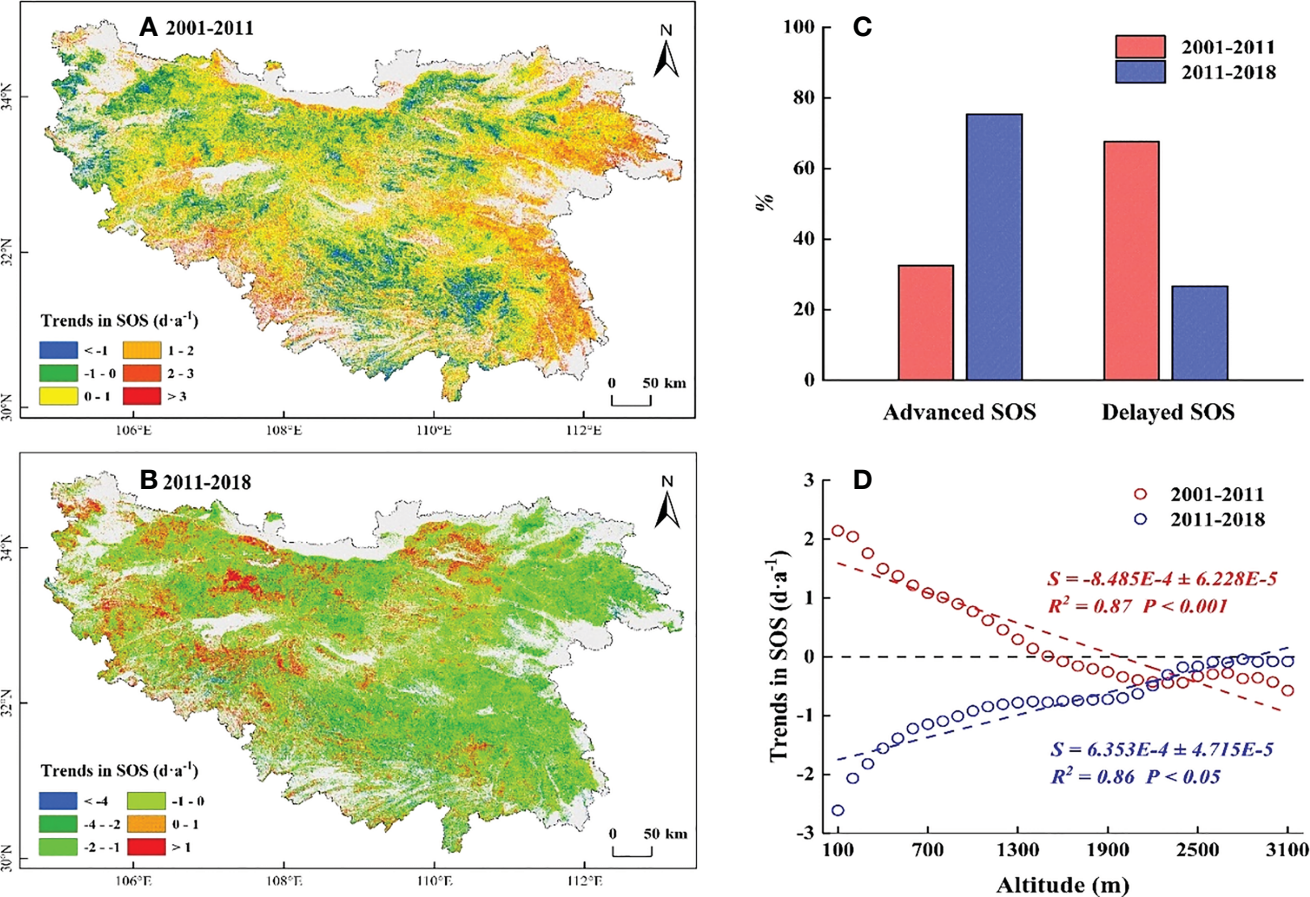
Spatial distribution of temporal trends in SOS during 2001-2011 **(A)** and 2011-2018 **(B)**. **(C)** The proportion of temporal trends in SOS. The redcolumns represent the proportion of SOS during 2001-2011,the blue columns represent the proportion of SOS during2011-2018. **(D)** The slope of trends in SOS along EG. The redline represent 2001-2011, the blue line represents 2011-2018.

The temporal trends in SOS before 2011 mainly showed a delayed trend (67.54%) that was distributed in the regions below 1500 m, and the magnitude decreased with increasing elevation (**Figures 4A–D**). Significant delayed (*P*< 0.05) were mainly distributed at low elevations in the eastern and southern regions (**Figure S2B**). While the advanced SOS (32.46%) was mainly distributed at high elevations above 1500 m, and the magnitude was increased (**Figures 4A–D**). Significant advanced (*P*< 0.05) were mainly distributed in the western, northern, and southern alpine regions (**Figure S2B**). These divergent trends showed a more uniform trend of SOS along EG in the QB.

However, the temporal trends in SOS after 2011 mainly showed an advanced trend (73.38%) at all elevations, and the magnitude decreased by 0.06 days·a ^-1^ for each 100 m increase (**Figures 4B–D**). Significant advanced (*P*< 0.05) were mainly distributed in the eastern and southern regions (**Figure S2C**). The advanced SOS (53.97%), which changed from a delayed SOS before 2011, was mainly distributed at low elevations (**Figure S3**), and the magnitude was stronger than that at high elevations. The difference in SOS gradually widened along EG. Shifts in the temporal trend of SOS at low elevations determined the direction of the uniform trend in SOS along EG.

### 3.3 Temporal trends in WT, ST and SP around 2011

**Figure 5:**
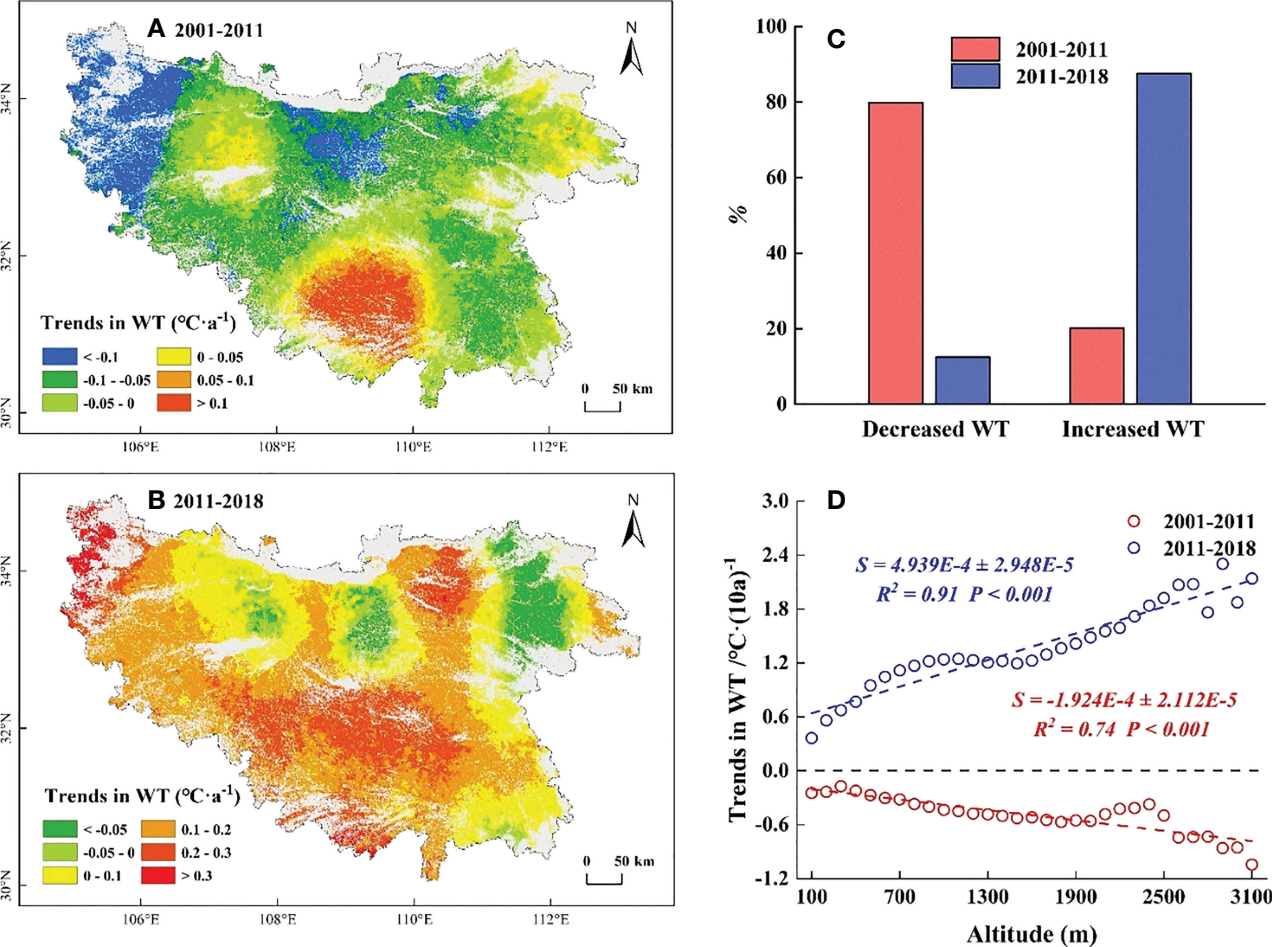
Temporal trends in winter temperature (WT) before and after 2011. **(A)** The temporal trends in WT during 2001-2011. **(B)** The temporal trends in WT during 2011-2018. **(C)** The proportion of temporal trends in WT. The red columns represent the proportion of WT during 2001-2011, the blue columns represent the proportion of WT during 2011-2018. **(D)** The slope of trends in WT along EG. The red line represents 2001-2011, the blue line represents 2011-2018.

The results of the trend analysis showed that the temporal trends in WT before 2011 mainly showed a cooling trend (79.82%), which was mainly distributed in marginal low elevation regions (**Figure 5A, C**). Significant decreased (*P*< 0.05) were mainly distributed in the eastern high elevation regions (**Figure S4A**). Along EG, the temporal trends in WT decreased by 0.02°C·(10a) ^-1^ for each 100 m increase (**Figure 5D**). After 2011, the temporal trends in WT mainly showed a warming trend (87.47%), which was mainly distributed in the marginal low elevation areas (**Figures 5B, C**). Significant increased (*P*< 0.05) were mainly distributed in the eastern and southern high elevation regions (**Figure S4D**). Along EG, the temporal trends in WT increased by 0.05°C·(10a) ^-1^ for each 100 m increase (**Figure 5D**).

**Figure 6:**
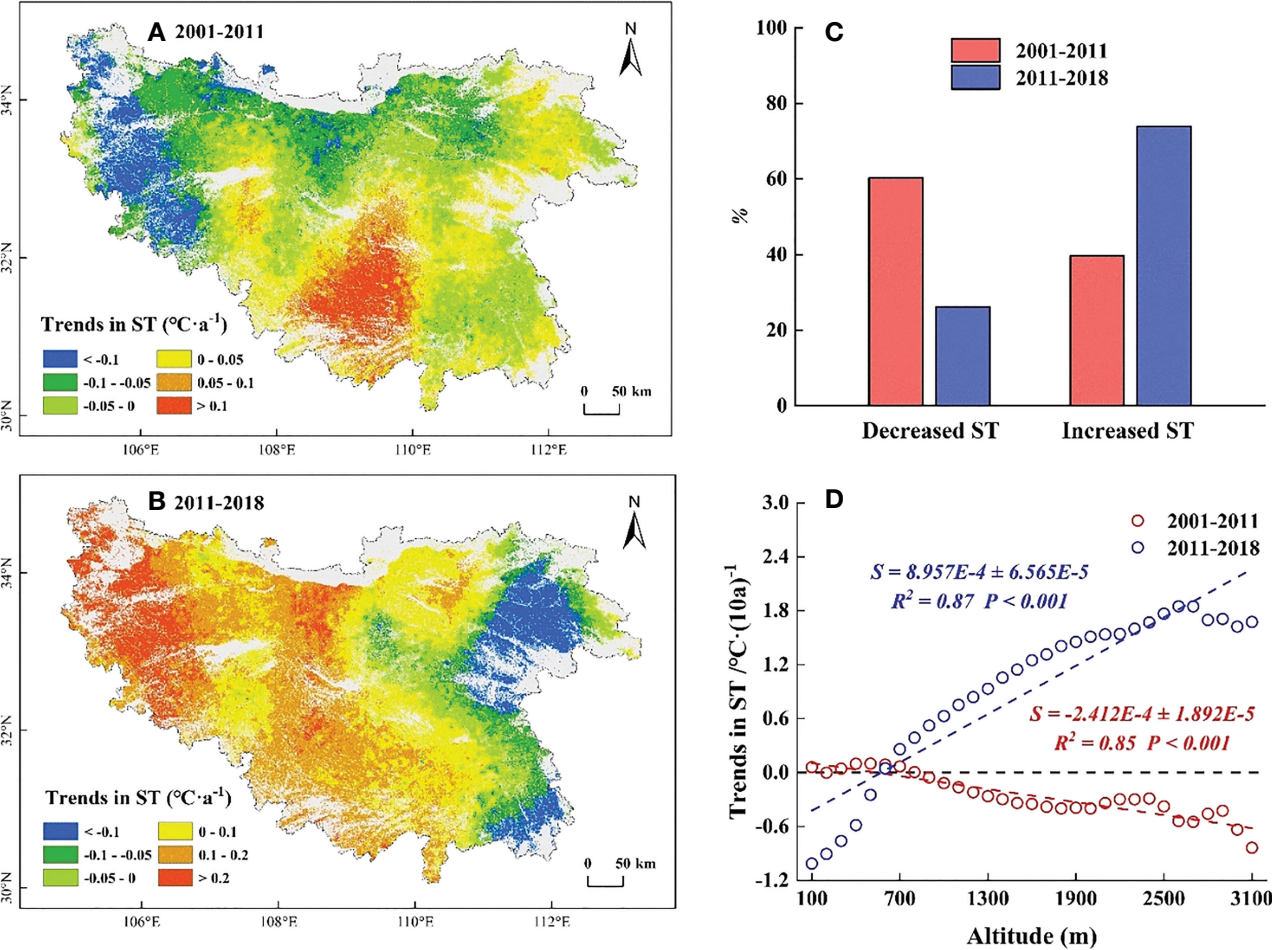
Temporal trends in spring temperature (ST) before and after 2011. **(A)** The temporal trends in ST during 2001-2011. **(B)** The temporal trends in ST during 2011-2018. **(C)** The proportion of temporal trends in ST. The red columns represent the proportion of ST during 2001-2011, the blue columns represent the proportion of ST during 2011-2018. **(D)** The slope of trends in ST along EG. The red line represents 2001-2011, the blue line represents 2011-2018.

The temporal trends in ST before 2011 mainly showed a cooling trend (60.26%), which was mainly distributed in high elevation regions (**Figures 6A, C**). Significant decreased (*P*< 0.05) were mainly distributed in the northern and eastern high elevation regions (**Figure S4B**). While the warming ST (39.74%) was concentrated in the marginal low elevation regions and the central Daba Mountains (**Figure 6A**). Along EG, the temporal trends in ST decreased by 0.02°C·(10a) ^-1^ for each 100 m increase, and the temporal trends in ST showed a slight increase below 800 m and then cooled (**Figure 6D**). After 2011, the temporal trends in ST mainly showed a warming trend (73.85%), which was mainly distributed in the central and western regions. While the cooling ST (26.15%) was concentrated in the eastern low elevation regions (**Figures 6B, C**), with a significant decreased (*P*< 0.05) in the northeastern regions (**Figure S4E**). Along EG, the temporal trends in ST increased by 0.09°C·(10a) ^-1^ for each 100 m increase, and the temporal trends in ST showed a decrease below 500 m and then warmed (**Figure 6D**).

**Figure 7:**
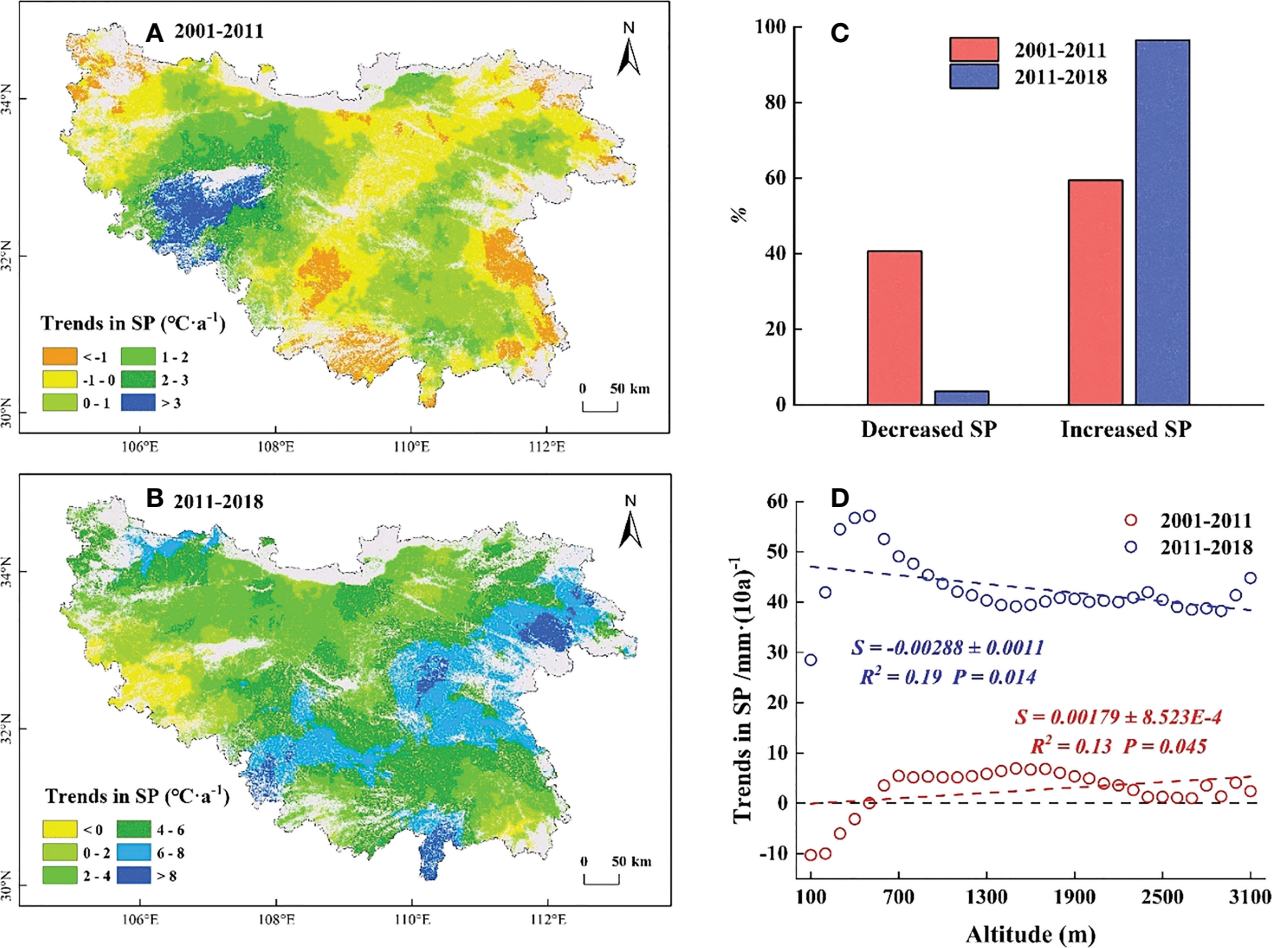
Temporal trends in spring precipitation (SP) before and after 2011. **(A)** The temporal trends in SP during 2001-2011. **(B)** The temporal trends in SP during 2011-2018. **(C)** The proportion of temporal trends in SP. The red columns represent the proportion of SP during 2001-2011, the blue columns represent the proportion of SP during 2011-2018. **(D)** The slope of trends in SP along EG. The red line represents 2001-2011, the blue line represents 2011-2018.

The temporal trends in SP before 2011 increased on average by 0.45 mm·a^-1^. Among them, the increased regions (59.4%) were mainly distributed at high elevations, while the decreased regions (40.6%) were distributed at marginally low elevations. The temporal trends in SP were of low amplitudes and mainly concentrated around 0-1 mm·a^-1^ (**Figures 7A, C**). Along EG, the temporal trends in SP increased by 0.18 mm·(10a)^-1^ for each 100 m increase (**Figure 7D**). After 2011, the temporal trends in SP increased by 4.41 mm·a^-1^ on average, which was approximately 10 times higher than that before 2011. Among them, the increased regions (96.45%) were mainly concentrated around 2-8 mm·a^-1^ (**Figures 7B, C**), with a significant increased (*P*< 0.05) in the eastern low elevation regions (**Figure S4F**). Along EG, the temporal trends in SP decreased by 0.29 mm·(10a)^-1^ for each 100 m increase (**Figure 7D**).

### 3.4 Relationship between SOS and its potential drivers

**Figure 8:**
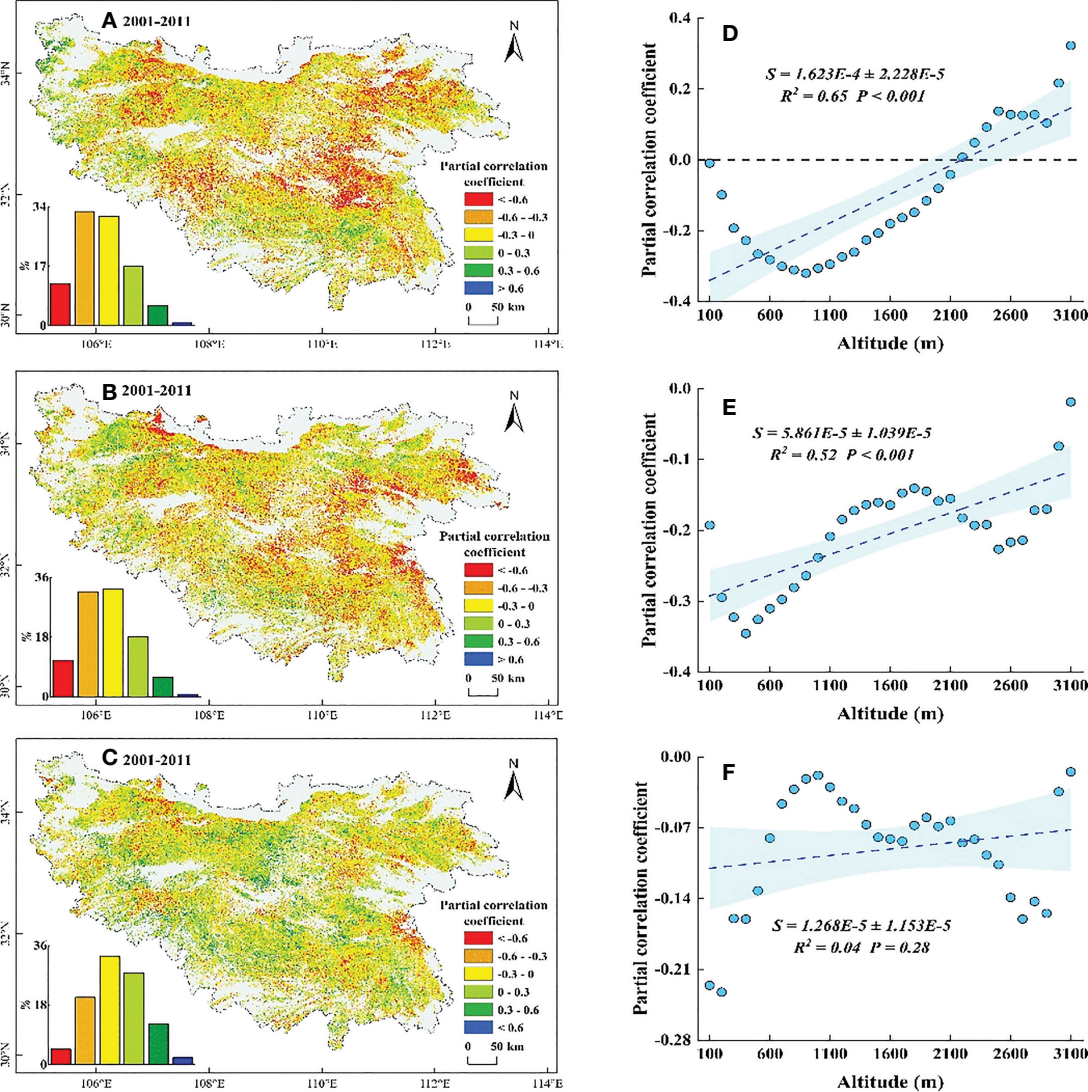
Partial correlation coefficient between SOS and its potential drivers during 2001-2011. **(A–C)** are correlations of SOS with WT, ST, and SP, respectively. **(D–F)** are the distributions of coefficients along EG corresponding to **(A–C)**, respectively. The shaded area indicates the 95% confidence interval.

Based on the results from the partial correlation analysis, we found that the SOS and WT were mainly negatively partially correlated (76.18%) in the QB before 2011, with a significant negative partial correlation (*P*< 0.05) in the central and eastern low elevation regions (**Figure S5A**), while the regions with a positive partial correlation (20.18%) were concentrated in the western and southern high elevation areas (**Figure 8A**). Along EG, the negative partial correlation coefficient gradually weakened with increasing elevation (below 2100 m), and the positive correlation coefficient continued to increase (above 2100 m) (**Figure 8D**). The SOS before 2011 showed a partial negative correlation with ST (75.36%), with a significant negative partial correlation (*P*< 0.05) in the central and eastern low elevation regions (**Figure S5B**), while the regions with a positive partial correlation coefficient (24.64%) were concentrated in the western and southern high elevation areas (**Figure 8B**). Along EG, the negative partial correlation coefficient significantly weakened (*P*< 0.001) with increasing elevation (**Figure 8E**). The SOS showed a partial negative correlation with SP (57.91%), with a significant negative partial correlation (*P*< 0.05) in the eastern low elevation regions and the western and southern high elevation regions (**Figure S5C**), while the regions with a positive partial correlation coefficient (42.09%) were distributed within all elevation gradients (**Figure 8C**). Along EG, the negative partial correlation coefficient did not change much (*P* = 0.28) with increasing elevation (**Figure 8F**).

**Figure 9:**
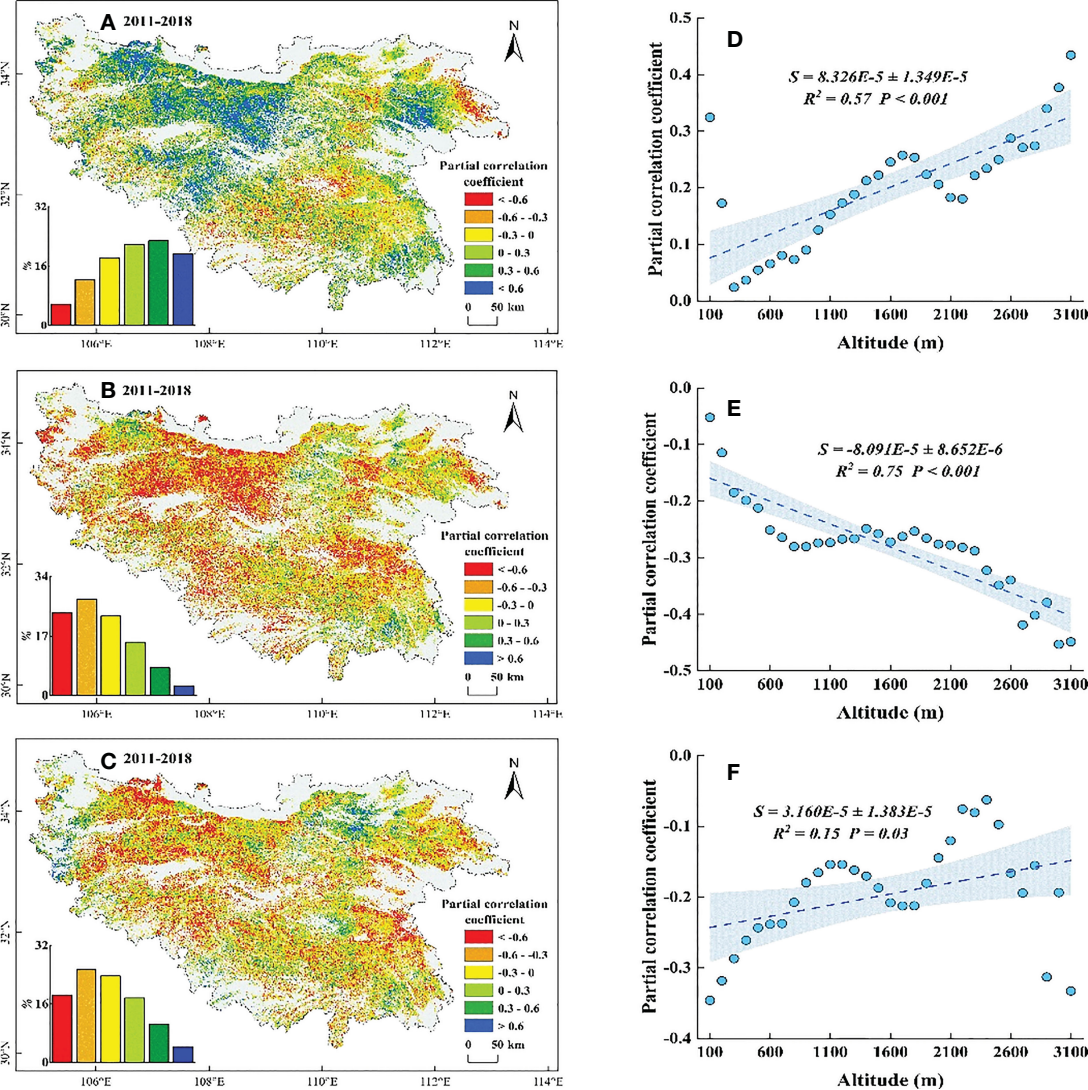
Partial correlation coefficient between SOS and its potential drivers during 2011-2018. **(A–C)** are correlations of SOS with WT, ST, and SP, respectively. **(D–F)** are the distributions of coefficients along EG corresponding to **(A–C)**, respectively. The shaded area indicates the 95% confidence interval.

For 2011-2018, the SOS and WT were mainly positively partially correlated (63.81%), with a significant positive partial correlation (*P*< 0.05) in the eastern low elevation regions and the central and western regions (**Figure S5D**), while the regions with a positive partial correlation (36.19%) were concentrated in the northeastern and southern high elevation areas (**Figure 9A**). Along EG, the positive partial correlation coefficient significantly increased (*P*< 0.001) with increasing elevation (**Figure 9D**). The SOS showed a partial negative correlation with ST (74%), with a significant negative partial correlation (*P*< 0.05) in the eastern low elevation regions and the central and western regions (**Figure S5E**), while the regions with a positive partial correlation coefficient (26%) were concentrated in the northeastern and southern high elevation areas (**Figure 9B**). Along EG, the negative partial correlation coefficient significantly increased (*P*< 0.001) with increasing elevation (**Figure 9E**). The SOS showed a partial negative correlation with SP (67.52%), with a significant negative partial correlation (*P*< 0.05) in the eastern low elevation regions and the western and southern high elevation regions (**Figure S5F**), while the regions with a positive partial correlation coefficient (32.48%) were sporadically distributed within all elevation gradients (**Figure 9C**). Along EG, the negative partial correlation coefficient significantly weakened (*P* = 0.03) with increasing elevation (**Figure 9F**).

## 4 Discussion

### 4.1 SOS response to driving factors

In this study, we found a decreased WT and a slightly increased ST below 500 m in the QB from 2001 to 2011, which somewhat provides more sufficient accumulation of chilling and forcing for the onset of spring phenology (Fu et al., 2015a; Ettinger et al., 2020; Pan et al., 2022). However, the SOS in this region was delayed (**Figure 4D**). Temperature alone did not sufficiently explain the SOS patterns in this region. Moreover, the reduced SP in this region (**Figure 7D**) somewhat limited the utility of water and heat conditions and offset the advanced SOS that would have been caused by the decreased WT and increased ST (Jewaria et al., 2021; Zheng et al., 2021), resulting in a delay in SOS. Hence, the decreased SP was the predominant controlling factor for the delayed SOS in this region. Similarly, at elevations of 500-1500 m, the decreased WT and increased SP somewhat facilitated the accumulation of chilling and the water demand for vegetation growth (Shen et al., 2015; Ettinger et al., 2020), which would have advanced the SOS. However, this advance would have likely been counteracted by reduced forcing as a result of the reduced ST (**Figure 6D**), which better explained why the SOS in this region was delayed (Piao et al., 2019; Ganjurjav et al., 2020). The decreased ST may have a stronger impact on SOS relative to the decreased WT and the increased SP. Meanwhile, at elevations above 1500 m, the decreased ST reduced the accumulation of forcing and delayed the SOS. Moreover, the decreased WT and the increased SP in this region (**Figures 5D**, **7D**) somewhat increased the accumulation of chilling and satisfied the water demand for the advanced SOS (Lin et al., 2022; Wang et al., 2022). The SOS response to reduced ST may have been less than that to the reduced WT and increased SP, resulting in the advanced SOS in this region. This showed that the joint control of the reduced WT and increased SP, rather than the ST, was the driving factor for the advanced SOS.

For 2011-2018, the increased WT and the reduced ST at elevations below 500 m were not conducive for more chilling and forcing accumulation (Yu et al., 2010; Wang et al., 2020a), which somewhat delayed the SOS. However, this delay was counteracted by the increased water demand as a result of the increased SP (**Figure 7D**) and led to the advance of SOS (Shen et al., 2015). The advanced SOS below 500 m was influenced by the increased SP rather than temperature. Similarly, at elevations above 500 m, the increased WT may reduce chilling accumulation and delay the SOS (Wang et al., 2020b; Jewaria et al., 2021). However, this delay was offset by the increased water demand and forcing accumulation (**Figures 6D**, **7D**), resulting in the advanced SOS (Piao et al., 2019; Li et al., 2021a). The effects of increased ST and SP on SOS were stronger than those of increased WT. The present results suggest that the SP also plays a crucial role in controlling the patterns of SOS along EG, and analyses conducted from the perspective of temperature alone may not fully elucidate the intrinsic mechanism of these patterns in SOS.

### 4.2 Effect of precipitation in controlling a more uniform SOS

Our research demonstrated that the magnitude of SOS response to SP gradually decreased and the response to temperature gradually increased with increasing elevation, which was consistent with previous studies in the QB (Liu et al., 2016; Chen et al., 2019). The SOS at high elevations, where the SP showed a consistent increase from 2001 to 2018 (**Figure 7D**), was less sensitive to SP. In other words, sufficient SP at high elevations resulted in less water stress on vegetation growth (Jennifer and Florence, 2018; Gupta et al., 2020), and larger amounts of SP would not advance SOS. Along with this was the potential for greater temperature sensitivity of SOS to maximise thermal benefits (Shen et al., 2015; Fu et al., 2021). This hypothesis was further supported by the weaker advance of SOS in the context of stronger precipitation after 2011 and the stronger partial correlation between SOS and temperature (**Figures 4D**, **9**). In contrast, at low elevations, where the SP showed a decrease from 2001 to 2011 (**Figure 7D**), maximised water usage led to the stronger sensitivity of SOS to SP (Shen et al., 2015; Chen et al., 2019). Stronger water stress led to a delayed SOS even under better temperature conditions (**Figures 5D**, **6D**). In addition, we speculated that the SOS would advance if more rainfall occurred after stronger water stress, even if the temperatures were less optimal. This hypothesis was confirmed by the significant advance of SOS at low elevations under the significant increase in SP after 2011 (**Figure 4D**, **Figure 7D**). This result showed that the temporal trends in SP played a crucial role in controlling the patterns of SOS at low elevations, which determined the direction of the uniform trend in SOS along EG.

Recent research has predicted that by the end of this century, the climate in east-central China will have a continuous warm-dry trend (Ma et al., 2019; Zhang et al., 2022), which may enhance water evaporation at low elevations in the QB. In addition, the structural overshoot due to the warm winter may further increase the water stress during the growing season (Bastos et al., 2020; Zhang et al., 2021b), thus strengthening the uniform trend of SOS along EG in the QB. Therefore, the sensitivity of SOS to precipitation may be further enhanced, and future changes in the spatiotemporal distribution of precipitation, rather than temperature, are likely to have a stronger control on the direction of the uniform trend in SOS. Since a more uniform SOS along EG may compromise the stability and serviceability of mountain ecosystems (Inouye et al., 2000; Lenoir et al., 2008; Wolf et al., 2017), it is crucial to improve the water-use efficiency of vegetation at low elevations. Previous studies have shown that a higher species diversity could notably enhance drought resistance (Grossiord, 2020; Liu et al., 2022), and different tree species have different strategies and abilities to cope with water stress (Anderegg et al., 2018; Li et al., 2020b). In the new round of ecological restoration projects, conversion of the current monoculture to mixed-species tree plantations and the planting of resilient tree species with a high water-use efficiency could relieve potential water stress in the future.

### 4.3 Comparison with other studies

In this study, we confirmed that the SOS extracted by the MOD13Q1 EVI can be used to accurately trace the spring phenology in the QB. This conclusion was predominantly based on the strong consistency with previous studies in related areas that were based on different data sources (**Table S2**). In addition, our findings demonstrated that the SOS showed a more uniform trend along EG between 2001 and 2018 in the QB. These divergent trends of SOS between low and high elevations led to a uniform trend of SOS along EG in the QB, which was consistent with the patterns of SOS in the Alps (Vitasse et al., 2018). However, the driving mechanisms of the more uniform SOS in the QB were different from those reported in the Alps. Vitasse et al. (2018), based on the hypothesis that temperature plays the dominant role, found that the reduced chilling accumulation at low elevations caused by the warming WT moderated the magnitude of SOS advance compared to high elevations. The SOS was more advanced at higher altitudes than at lower altitudes, resulting in a more uniform trend of SOS along EG. In this study, we added the effects of SP and found that the SP, rather than temperature, determined the direction of the uniform trend in SOS by controlling the SOS patterns at low elevations. These differences may have been due to potential mechanisms playing diverse roles across different areas.

In addition, the results demonstrated negative relationships between ST, SP and SOS during 2001-2018 and a positive relationship between WT and SOS between 2011 and 2018, which was consistent with most previous phenological studies (Piao et al., 2006; Zheng et al., 2021). However, the WT was negatively correlated with SOS below 2100 m during 2001-2011 and the cooling WT delayed the SOS in the QB. The simultaneous reduction of ST between 500 and 2100 m and the SP below 500 m may have counteracted the advance of SOS caused by decreased WT (Cong et al., 2017; Asse et al., 2018), possibly explaining why the SOS was delayed under a cooling WT.

In contrast to our results, Gao et al. (2019) found that there were no prevalent trends of elevational homogenization of SOS in most regions worldwide over the last 30 years. This discrepancy was likely due to the difference in the main drivers of vegetation growth at different spatial scales. For example, the stronger water stress in arid areas results in a greater sensitivity of SOS to precipitation, but the sensitivity of SOS to temperature and precipitation may change at a larger scale (Cleverly et al., 2016; Cong et al., 2021). In addition, the magnitudes and even the directions of the response to climate change are largely different among vegetation types (Lesica and Kittelson, 2010; Fu et al., 2015b). The proportion of vegetation types at different spatial scales varies, which may lead to differences in the variation patterns of SOS.

### 4.4 Uncertainty analysis

First, using different abrupt change tests may lead to different results. We used the M-K test, sliding t-test, and quadratic curve fitting test to detect abrupt changes in the slope of SOS along EG from 2001 to 2018 (**Figure S6**). We found that the abrupt change years were approximately 2003, 2008, and 2011 in the M-K test, sliding t test, and quadratic curve fitting test, respectively. Moreover, previous research has shown that the vegetation coverage reversed in 2010 in the QB during 2001-2014 (Liu et al., 2016). Chen et al. (2021) found that 2010 was the significant acceleration point for gross and net primary production variations in China during 2001-2018. Therefore, using the quadratic curve fitting test, we defined 2011 as an abrupt change year. Second, since the CMFD dataset was reanalysis data, the temperature and precipitation data already contained elevation information (He et al., 2020). Downscaling by selecting the EVI and elevation as influencing factors may overestimate the effect of elevation on temperature and precipitation, which somewhat impacts the downscaling results. Finally, different vegetation types have different responses to climate change. As the dividing line between north subtropical and temperate forests, vegetation types in the QB are diverse and have an obvious altitudinal differentiation (Deng et al., 2019; Qi et al., 2021). Therefore, the patterns of SOS along EG on vegetation types and the north and south slopes need to be further studied.

## 5 Conclusions

The variation patterns of SOS along EG in the QB from 2001 to 2018 were explored. We found that the SOS showed a more uniform trend along EG in the QB. Furthermore, this uniform trend of SOS along EG was not completely continuous and reversed around 2011. The ST and SP were, in general, negatively partially correlated with SOS, and WT was positively partially correlated with SOS (except for regions below 2100 m during 2001-2011). Before 2011, the decreased ST and SP led to a delayed SOS at low elevation and the increased SP and decreased WT led to an advanced SOS at high elevation. These opposite SOS trends at high and low elevations led to a more uniform SOS along EG. After 2011, increased SP and ST led to a much greater advance of SOS at low elevations than at high elevations, resulting in a gradual widening of the difference in SOS along EG. Moreover, the temporal trends in SP played a crucial role in controlling the SOS patterns at low elevations, which determined the direction of the uniform trend in SOS along EG. Our study deepens the understanding of the altitudinal sensitivity of SOS under climate change and provides a theoretical basis for regions that experience a uniform trend of SOS along EG to develop appropriate ecological measures to mitigate its adverse effects.

## Data availability statement

The original contributions presented in the study are included in the article/**Supplementary Material**, further inquiries can be directed to the corresponding author.

## Author contributions

JL and JZ conceived and designed this study. BL, DY, and JG provided guidance on research methods and content, WH and RT assisted with the data analysis, JL wrote the first draft of the manuscript. All authors contributed to manuscript revision, read and approved the submitted version.
