# Supplementary material for: Important role of precipitation in controlling a more uniform spring phenology in the Qinba Mountains, China
Source: Front Plant Sci. 2023 Feb 8;14:1074405. doi: 10.3389/fpls.2023.1074405 (PMC9945530; doi:10.3389/fpls.2023.1074405)
Supplement: Supplementary file 1 [file DataSheet_1.pdf]

Supplementary Materials for

Important role of precipitation in controlling a more uniform spring phenology in the Qinba Mountains, China

Jianhao Li[1], Jingyun Guan[1,2], Wangqiang Han[1], Ruikang Tian[1], Binbin Lu[3], Danlin Yu[4], Jianghua Zheng[1*]

[1] College of Geography and Remote sensing Sciences, Institute of Arid Ecology and Environment, Key Laboratory of Oasis Ecology, Xinjiang University, Urumqi 830046, China

[2] College of Tourism, Xinjiang University of Finance & Economics, Urumqi 830012, China

[3] School of Remote Sensing and Information Engineering, Wuhan University, Wuhan 430079, China

[4] Department of Earth and Environmental Studies, Montclair State University, New Jersey 07043, USA

** Corresponding author*:

Jianghua Zheng

College of Geography and Remote sensing Sciences, and Key Laboratory of Oasis Ecology under Ministry of Education Xinjiang University, Urumqi 830046, China

Email: zheng.jianghua@xju.edu.cn

This PDF file includes:

Figs. S1 to S6

Tables S1 to S2

Table S1. The slope of SOS along EG in the QB from 2001 to 2018.

Year	Slope of SOS along EG	R^2
2001	S = 0.02038 ± 8.58018E-4	0.95***
2002	S = 0.01989 ± 7.37398E-4	0.96***
2003	S = 0.01621 ± 6.35332E-4	0.96***
2004	S = 0.01974 ± 7.11802E-4	0.96***
2005	S = 0.01359 ± 4.16969E-4	0.97***
2006	S = 0.01738 ± 3.81774E-4	0.99***
2007	S = 0.01698 ± 5.54988E-4	0.97***
2008	S = 0.01177 ± 4.50813E-4	0.96***
2009	S = 0.01351 ± 7.45447E-4	0.92***
2010	S = 0.013 ± 5.94711E-4	0.94***
2011	S = 0.01245 ± 8.44703E-4	0.89***
2012	S = 0.01161 ± 5.96086E-4	0.93***
2013	S = 0.01403 ± 5.14453E-4	0.96***
2014	S = 0.01637 ± 9.63645E-4	0.91***
2015	S = 0.01205 ± 0.00103	0.82***
2016	S = 0.0185 ± 5.73025E-4	0.97***
2017	S = 0.01746 ± 4.2414E-4	0.98***
2018	S = 0.01226 ± 6.82392E-4	0.91***

Table S2. Comparison of our results with previous studies in related regions in China.

Area	SOS/d	Data form	Time scale	Reference
Temperate China	50 - 180	SPOT	1999 - 2009	Cong et al., 2020
Subtropical China	50 - 140	SIF	2002 - 2017	Li et al., 2021
Yangtze River Basin	70 - 160	GIMMS	1982 - 2015	Yuan et al., 2019
QB Mountains	60 - 110	AVHRR, MODIS, VIIRS	1981 - 2016	Li et al., 2020
Qinling-Huanghuai Plain	67 - 116	MOD09Q1	2002 - 2020	Wang et al., 2022
Qinling Mountains	83 - 121	MOD13A2	2001 - 2019	Guo et al., 2021
Qinling Mountains	73 - 113	MOD13Q1	2001 - 2018	Li et al., 2021
Xian station	97	Ground station	1985 - 2015	Deng et al., 2017
Xingangshan, Jiangxi Province, China	71 - 113	Ground station	2009 - 2010	Du et al., 2020
QB mountains	73 - 105	MOD13Q1	2001 - 2018	This study

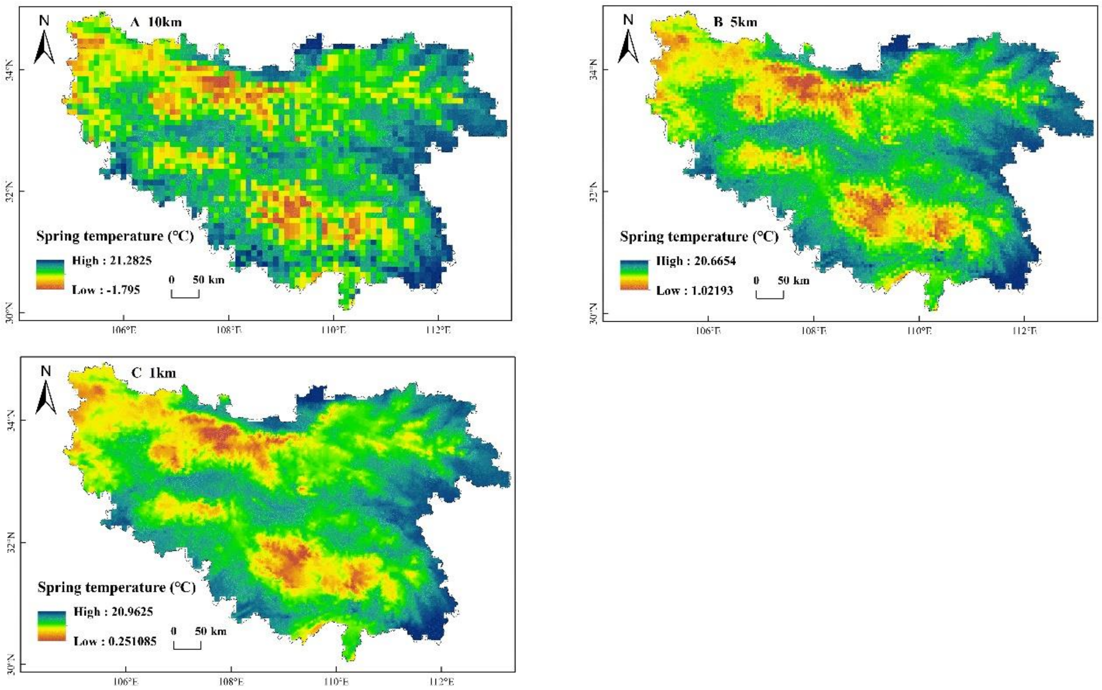

Fig. S1. Spatial downscaling of climate data using geographically weighted regression in three levels.

The spring temperature of 2001 was used as an example.

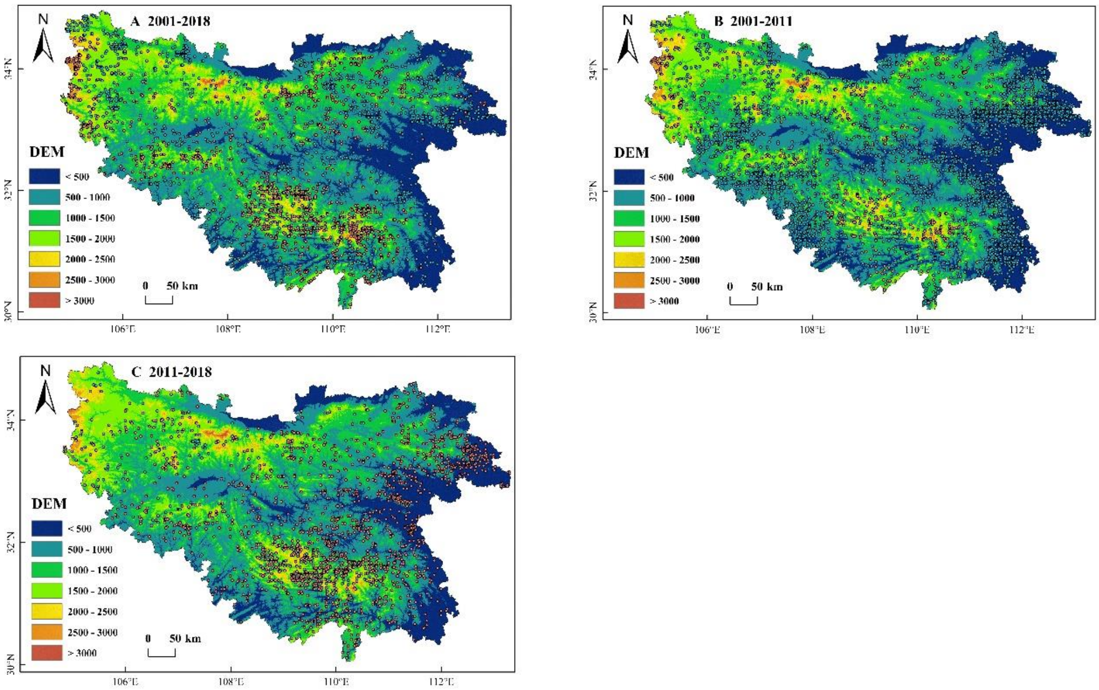

Fig. S2. Temporal trends in SOS passing significance tests ($P < 0.05$). The red and blue dots indicate significantly advanced and delay , respectively.

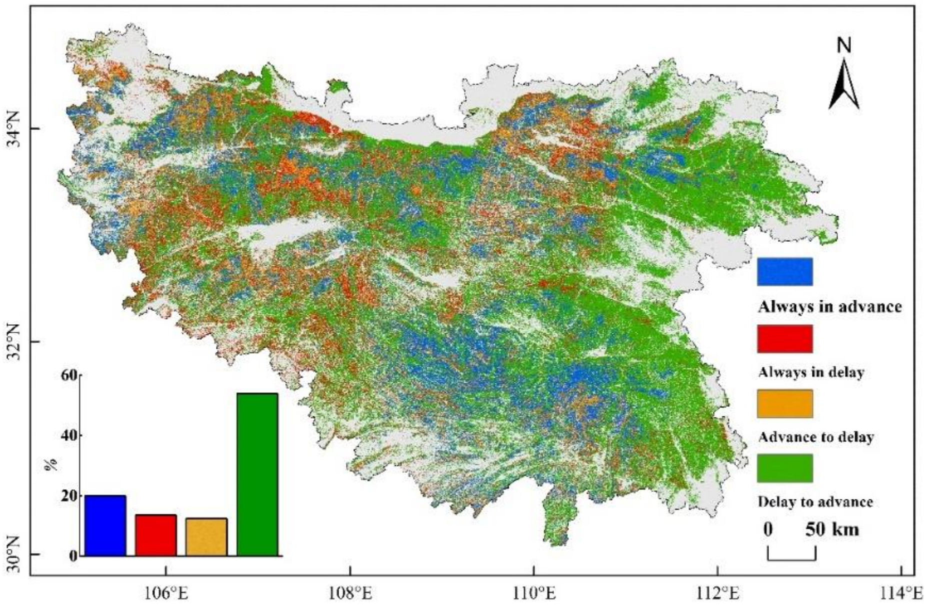

Fig. S3. Changes in temporal trends in SOS after 2011.

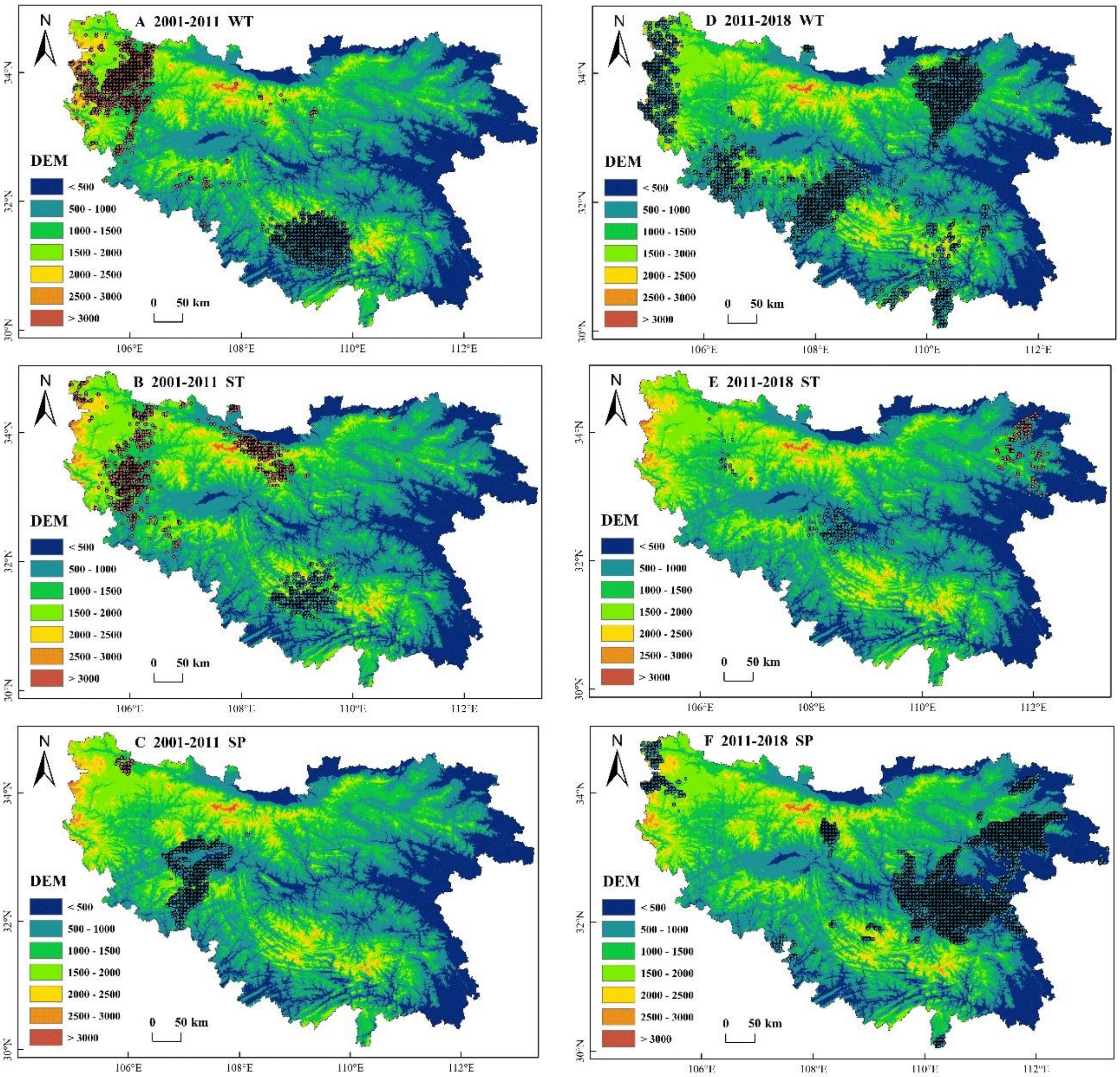

Fig. S4. Temporal trends in WT、ST and SP passing significance tests ($P < 0.05$). The red and blue dots indicate significantly decreased and increased , respectively.

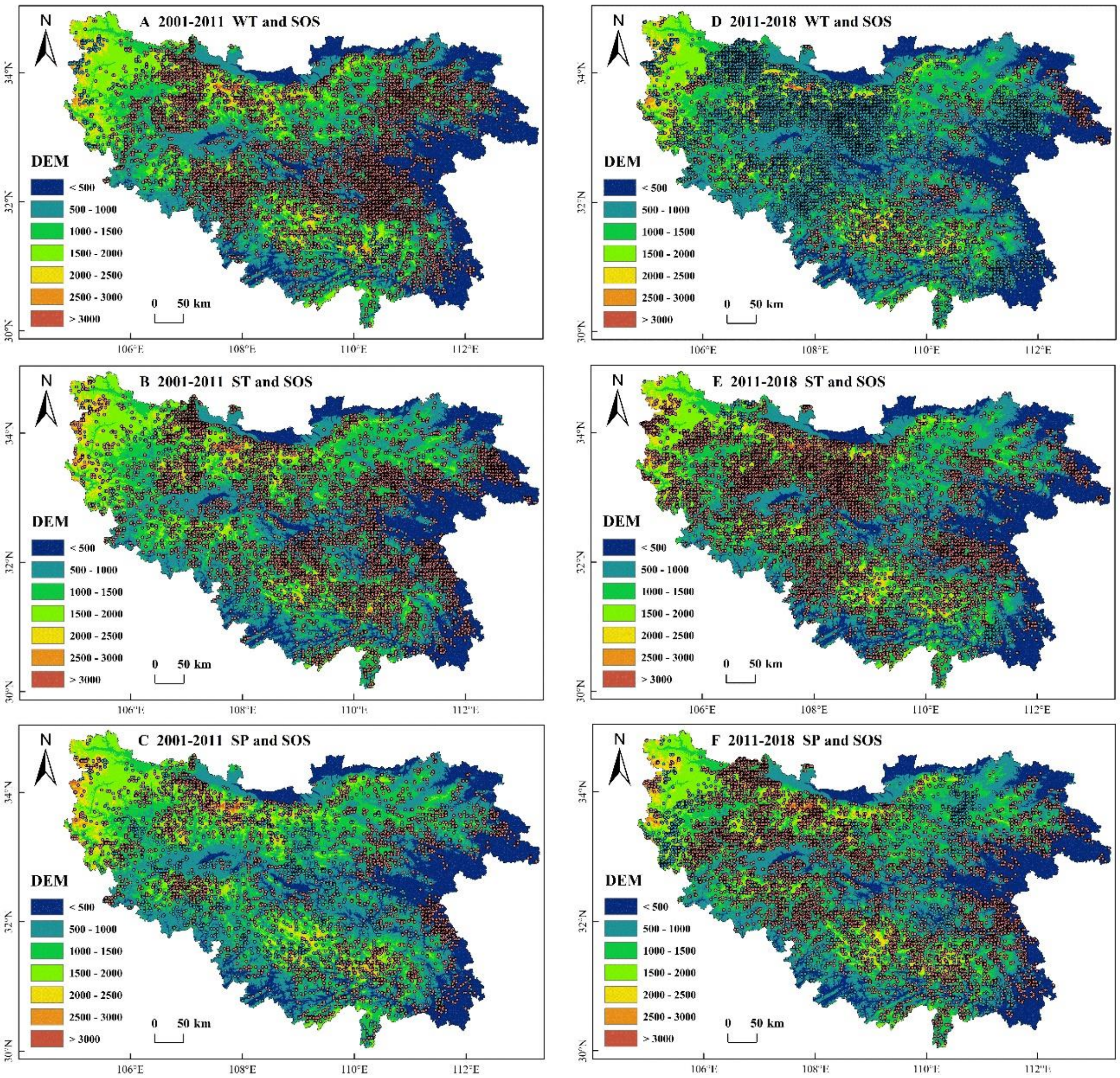

Fig. S5. Partial correlation coefficient between WT、ST and SP and SOS passing significance tests ($P < 0.05$). The red and blue dots indicate significantly negative and positive correlations , respectively.

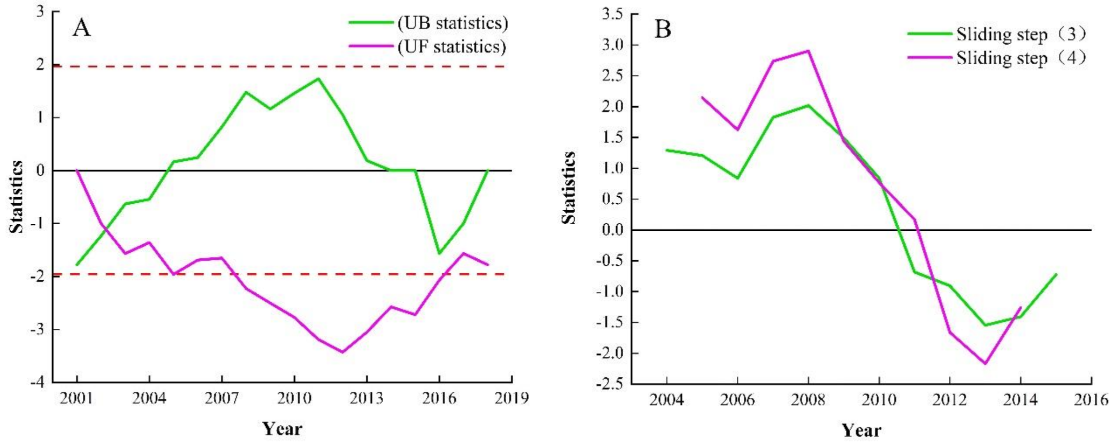

Fig. S6. Abrupt point test for slope of SOS along EG. A and B are based on the MK test and the sliding t-test. , respectively.
